# Cannabinoid Receptor 2 Signalling Bias Elicited by 2,4,6-Trisubstituted 1,3,5-Triazines

**DOI:** 10.3389/fphar.2018.01202

**Published:** 2018-11-20

**Authors:** Caitlin R. M. Oyagawa, Sara M. de la Harpe, Yurii Saroz, Michelle Glass, Andrea J. Vernall, Natasha Lillia Grimsey

**Affiliations:** ^1^Department of Pharmacology and Clinical Pharmacology, School of Medical Sciences, Faculty of Medical and Health Sciences, The University of Auckland, Auckland, New Zealand; ^2^Centre for Brain Research, School of Medical Sciences, Faculty of Medical and Health Sciences, The University of Auckland, Auckland, New Zealand; ^3^School of Pharmacy, University of Otago, Dunedin, New Zealand

**Keywords:** cannabinoid receptor 2 (CB_2_), G protein-coupled receptor, signalling bias, signalling, synthetic cannabinoid, drug design, medicinal chemistry, immune therapeutics

## Abstract

Cannabinoid receptor 2 (CB_2_) is predominantly distributed in immune tissues and cells and is a promising therapeutic target for modulating inflammation. In this study we designed and synthesised a series of 2,4,6-trisubstituted 1,3,5-triazines with piperazinylalkyl or 1,2-diethoxyethane (PEG2) chains as CB_2_ agonists, all of which were predicted to be considerably more polar than typical cannabinoid ligands. In this series, we found that triazines containing an adamantanyl group were conducive to CB_2_ binding whereas those with a cyclopentyl group were not. Although the covalent attachment of a PEG2 linker to the adamantyl triazines resulted in a decrease in binding affinity, some of the ligands produced very interesting hCB_2_ signalling profiles. Six compounds with notable hCB_2_ orthosteric binding were functionally characterised in three pathways; internalisation, cyclic adenosine monophosphate (cAMP) and ERK phosphorylation (pERK). These were predominantly confirmed to be hCB_2_ agonists, and upon comparison to a reference ligand (CP 55,940), four compounds exhibited signalling bias. Triazines **14** (UOSD017) and **15** were biased towards internalisation over cAMP and pERK, and **7** was biased away from pERK activation relative to cAMP and internalisation. Intriguingly, the triazine with an amino-PEG2-piperazinyl linker (**13** [UOSD008]) was identified to be a mixed agonist/inverse agonist, exhibiting apparent neutral antagonism in the internalisation pathway, transient inverse agonism in the cAMP pathway and weak partial agonism in the pERK pathway. Both the cAMP and pERK signalling were pertussis toxin (PTX) sensitive, implying that **13** is acting as both a weak agonist and inverse agonist at CB_2_ via Gα_i/o_. Compound **10** (UOSD015) acted as a balanced high intrinsic efficacy agonist with the potential to produce greater hCB_2_-mediated efficacy than reference ligand CP 55,940. As **10** includes a Boc-protected PEG2 moiety it is also a promising candidate for further modification, for example with a secondary reporter or fluorophore. The highest affinity compound in this set of relatively polar hCB_2_ ligands was compound **16**, which acted as a slightly partial balanced agonist in comparison with CP 55,940. The ligands characterised here may therefore exhibit unique functional properties *in vivo* and have the potential to be valuable in the future development of CB_2_-directed therapeutics.

## Introduction

Cannabinoid receptors 1 (CB_1_) and 2 (CB_2_) belong to the G protein-coupled receptor (GPCR) superfamily, and are the two cannabinoid receptors that have been cloned and characterised to date. CB_1_ is highly expressed in the central nervous system (CNS) and its function is imperative in many physiological processes including regulation of mood and appetite, pain perception, learning and memory, and motor control (Marsicano and Lutz, 2006; Kano et al., 2009; De Laurentiis et al., 2014), whereas CB_2_’s distribution is predominantly in immune tissues and cells (Bouaboula et al., 1993; Galiegue et al., 1995), and it accordingly plays an important role in immunomodulation. At the molecular level, CB_2_ couples to and signals downstream of Gα_i_, leading to inhibition of cyclic adenosine monophosphate (cAMP) production via adenylate cyclase and phosphorylation of ERK1/2 (pERK) (Felder et al., 1995; Bouaboula et al., 1996). Downstream effects of CB_2_ activation include the induction of natural killer cell migration (Kishimoto et al., 2005), regulation of the differentiation of B and T lymphocytes (Ziring et al., 2006), and modulation of cytokine release (Cencioni et al., 2010; Correa et al., 2011). CB_2_ expression in the CNS has been reported, particularly in microglia, where its activation was shown to produce anti-inflammatory effects (Ma et al., 2015). CB_2_ has also been identified in other areas of the body including in osteoblasts and osteoclasts (Ofek et al., 2006), and in the gastrointestinal tract (Atwood and Mackie, 2010), and has thus been indicated as a potential therapeutic target in chronic inflammatory conditions such as osteoporosis (Karsak et al., 2005; Bab and Ofek, 2011) and inflammatory bowel disease (Wright et al., 2005, 2008). There is also potential for CB_2_ therapeutic intervention in cancers, as receptor and endogenous ligand upregulation has been associated with tumour aggressiveness in some studies (Velasco et al., 2012). Furthermore, due to its largely peripheral distribution, activation of CB_2_ is not associated with the psychotropic effects that result from CB_1_ activation, further supporting it as a promising therapeutic target. As such, a focus of recent research efforts has been in developing and evaluating CB_2_-selective ligands as well as peripherally-restricted CB_1_ and CB_2_ ligands.

Phyto- and endo-cannabinoids, as well as the vast majority of synthetic cannabinoids produced to date, possess high water-octanol partition coefficients (LogP) and are thus inherently highly lipophilic with generally poor pharmacokinetic properties from a drug development perspective (e.g., oral bioavailability) (McGilveray, 2005; Huestis, 2007). A wide range of scaffolds have been reported as CB_2_ agonists (reviewed by Morales et al., 2016), including those with reduced lipophilicity in comparison with “traditional” cannabinoids which may have improved properties in the context of pharmacological development (e.g., Odan et al., 2012; Yrjölä et al., 2015). CB_2_ ligands with novel physicochemical properties may also possess unique binding modalities, particularly in comparison with a prevailing theory that ligands can enter the CB_2_ binding pocket via the lipid bilayer (Hurst et al., 2010). In addition, polar ligands may have reduced access to intracellular receptor populations due to a reduced propensity to cross the plasma membrane in comparison with more lipophilic ligands (Liu et al., 2011). Indeed, CB_2_ has been reported to be expressed both at the cell surface and intracellularly (Kleyer et al., 2012; Brailoiu et al., 2014; Castaneda et al., 2017), and intracellular CB_2_ may be able to activate distinct signalling responses from surface CB_2_ (Brailoiu et al., 2014). Thus ligands that differentiate between these populations would have the potential to induce differential signalling patterns in comparison with lipophilic ligands that can interact with both surface and intracellular receptors. Furthermore, an increase in CB_2_ ligand polarity can restrict blood–brain barrier permeability and therefore entry to the CNS (van der Stelt et al., 2011; Odan et al., 2012), which could also be therapeutically advantageous.

An additional motivation in designing ligand scaffolds with increased polarity and/or hydrophilic chains is in preparation for conjugation to fluorophores, i.e., fluorescent ligands, and other functional groups which can be valuable tools in characterising receptor localisation and function (Stoddart et al., 2015). As a prerequisite for making such ligands, it is necessary to investigate which chemical groups on a scaffold can be modified whilst retaining affinity and functional activity at the receptor of interest.

Recently, a set of 2,4,6-trisubstituted 1,3,5-triazine analogues were reported as potent CB_2_ agonists (Yrjölä et al., 2013). These analogues are highly lipophilic, and as such Yrjölä et al. (2015) commented that these compounds may have limited applicability *in vivo*. To address this, Yrjölä et al. (2015) synthesised a revised set of 2,4,6-trisubstituted 1,3,5-triazine analogues with polar functional groups with overall decreased lipophilicity and increased water solubility. In this study we built further upon this scaffold and synthesised a set of compounds based on the most potent CB_2_ full agonist reported in the aforementioned study (Yrjölä et al., 2015), **15** in the present manuscript. We synthesised and characterised thirteen 2,4,6-trisubstituted 1,3,5-triazine analogues comprised of five previously published (**1**, **2**, **7**, **15**, **16**) and eight novel triazines (**5**, **6**, **9**–**14**), some of which include linkers for potential fluorophore attachment (Table 1). Compounds which measurably bound to hCB_2_ (above a pre-defined cut-off) were assessed for their function via *in vitro* hCB_2_ assays (internalisation, cAMP, and pERK). The responses in these pathways were then analysed for signalling bias.

**Table 1:** Structures and hCB_2_ binding affinities for CP 55,940 and 2,4,6-trisubstituted 1,3,5-triazine analogues.

	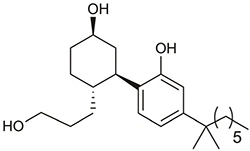 CP 55,940	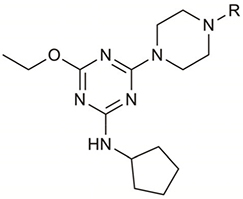 2, 5, 6		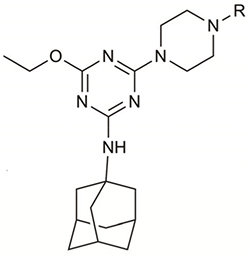 7, 9-16		
Compound # (name)	R group	cLogP	cLogD_7.4_	CB_2_ binding extent at 10 μM (%)^Δ^ (±SEM)	CB_2_ pK_d_ or pK_i_^∗^ (±SEM)	CB_1_ binding extent at 10 μM (%)^Δ^ (±SEM)

CP 55,940	(Structure above)	5.57	5.57	100.0 (0.0)	8.89 (0.07)	100.0 (0.0)
1^+^	(Refer to Scheme 1)	2.67	2.67	–8.1 (4.7)	N.D.	N.D.

2^∧^	H	2.18	0.90	5.8 (2.7)	N.D.	N.D.

**5** (UOSD005)	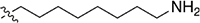	3.05	–0.16	16.6 (4.5)	N.D.	N.D.

**6** (UOSD009)	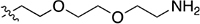	1.42	–0.71	15.6 (5.0)	N.D.	N.D.

7^∧^	H	2.71	1.43	85.6 (3.4)	6.38 (0.16)	13.7 (4.1)

**9** (UOSD016)	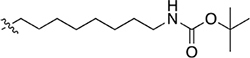	4.86	4.37	51.9 (9.8)	N.D.	N.D.

**10** (UOSD015)	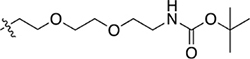	3.24	3.14	91.8 (3.6)	6.18 (0.10)	16.7 (7.8)

**11** (UOSD007)	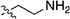	2.28	0.32	40.1 (4.7)	N.D.	N.D.

**12** (UOSD010)	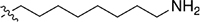	3.58	0.37	43.5 (4.5)	N.D.	N.D.

**13** (UOSD008)	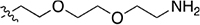	1.95	–0.18	81.1 (2.2)	5.74 (0.06)	29.6 (7.6)

**14** (UOSD017)	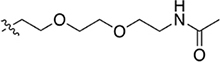	1.78	1.68	76.2 (1.8)	5.75 (0.02)	–18.8 (11.4)

15^∧^	Me	3.07	2.92	92.9 (2.0)	8.37 (0.18)	62.5 (2.6)

16^∧^	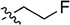	3.23	3.22	95.6 (3.3)	8.79 (0.03)	81.7 (2.7)


*N.D., Not determined. ^∗^pK_*d*_ for CP 55,940, or pK_*i*_ for all other compounds. ^+^First reported in Yrjölä et al. (2013). ^∧^First reported in Yrjölä et al. (2015). ^Δ^Binding extent expressed as percentage of [^*3*^H]-CP 55,940 displacement.*

## Materials and Methods

### Chemistry

Chemicals were purchased from Sigma Aldrich (St Louis, MO, United States), Merck (Darmstadt, Germany), AK Scientific (Union City, CA, United States), or Ark Pharm, Inc. (Libertyville, IL, United States). Reactions were carried out at room temperature unless otherwise stated. Flash silica gel column chromatography was performed with 40–63 μm silica and thin layer chromatography with 0.2 mm aluminium-backed silica gel plates 60 F_254_ using UV light, ninhydrin and/or KMnO_4_. An Agilent 1260 Infinity system (Santa Clara, CA, United States) was used for reverse phase high-performance liquid chromatography (RP-HPLC), with a C8 5 μm (150 × 10 mm) semi-preparative or C8 5 μm (150 × 4.6 mm) analytical column (YMC, Kyoto, Japan). RP-HPLC solvents were A: H_2_O (0.05% TFA) and B: 9:1 MeCN:H_2_O (0.05% TFA). Analytical RP-HPLC retention times are given using – 5% solvent B 1 min, gradient of 5–95% solvent B 1–27 min, 95% solvent B 27–28 min, gradient of 95–5% solvent B 28–30 min, 5% solvent B 30–34 min. Purities were assessed by analytical RP-HPLC analysis at 254 and 380 nm. Compound purities are given for all test compounds as a % purity via peak integration at 254 nm. TFA salts of RP-HPLC purified compounds were neutralised using an Amberlyst A21 ion exchange resin before biological testing. High resolution electrospray ionisation mass spectroscopy (HRMS-ESI) was carried out using a Bruker micrOTOF mass spectrometer (Bruker, Billerica, MA, United States). NMR spectra were obtained using a Varian 400-MR or 500 MHz AR premium shielded spectrometer (Varian, Palo Alto, CA, United States). Chemical shifts are listed in ppm (δ), calibrated using residual undeuterated solvent, and coupling constants (*J*) are recorded in hertz (Hz). clogP and clogD_7.4_ were calculated in MarvinSketch (version 17.6.0, ChemAxon) using the “ChemAxon” calculation method and the default electrolyte concentration (Cl^-^ 0.1 mol/dm^3^, Na^+^ K^+^ 0.1 mol/dm^3^).

4-Chloro-*N*-cyclopentyl-6-ethoxy-1,3,5-triazin-2-amine **1** and *N*-cyclopentyl-4-ethoxy-6-(piperazin-1-yl)-1,3,5-triazin-2-amine **2** were prepared as previously reported (Yrjölä et al., 2013, 2015). **1** and **2** were characterised by NMR spectroscopy and HRMS (data not shown), and sample purity of test compounds was 97.3 and 100%, respectively.

#### *tert*-Butyl *N*-(6-(4-(4-(Cyclopentylamino)-6-Ethoxy-1,3,5-Triazin-2-yl)Piperazin-1-yl)Hexyl)Carbamate (3)

A mixture of **2** (0.12 g, 0.41 mmol), *tert*-butyl *N*-(6-bromohexyl) carbamate (0.12 g, 0.41 mmol), K_2_CO_3_ (0.17 g, 1.23 mmol), and NaI (2 mg) in dioxane (30 mL) was refluxed for 24 h. The solvent was removed under reduced pressure and the residue diluted with CH_2_Cl_2_ and washed with water. The organic layer was dried with MgSO_4_, the solvent concentrated under reduced pressure and purified by silica gel flash chromatography (100% EtOAc) to give **3** (0.15 g, 0.30 mmol, 72% yield) as a yellow oil. ^1^H NMR (400 MHz, CDCl_3_) δ 1.32 – 1.72 (m, 17H), 1.44 (s, 9H), 2.00 (m, 2H), 2.38 – 2.55 (br m, 6H), 3.11 (m, 2H), 3.84 (m, 4H), 4.29 (m, 2H), 4.51 (br s, 1H), 4.95 (br s, 1H). ^13^C NMR (101 MHz, CDCl_3_) δ 14.5, 23.7, 26.3, 26.6, 27.1, 28.4, 30.0, 33.2, 40.4, 42.8, 52.5, 52.9, 58.5, 62.2, 78.9, 156.0, 165.9, 166.5, 170.4. HRMS (m/z): [M + H]^+^ calcd. for C_25_H_46_N_7_O_3_, 492.3657; found, 492.3624.

#### *tert*-Butyl *N*-(2-(2-(2-(4-(4-(Cyclopentylamino)-6-Ethoxy-1,3,5-Triazin-2-yl)Piperazin-1-yl)Ethoxy) Ethyl)Carbamate (4)

**2** (85.0 mg, 0.29 mmol) and *tert*-butyl *N*-{2-[2-(2-bromoethoxy) ethoxy]ethyl}carbamate (85.0 mg, 0.29 mmol) were reacted according to the procedure for **3** and purified by silica gel flash chromatography (5% MeOH/CH_2_Cl_2_) to give **4** (100.0 mg, 0.19 mmol, 66% yield) as a pale yellow oil. ^1^H NMR (400 MHz, CDCl_3_) δ 1.35 (m, 3H), 1.40 – 1.46 (m, 11H), 1.61 (m, 2H), 1.70 (m, 2H), 2.00 (m, 2H), 2.77 – 2.90 (br m, 6H), 3.31 (m, 2H), 3.53 (t, *J* = 5.2 Hz, 2H), 3.61 (m, 4H), 3.81 (m, 2H), 3.99 (m, 4H), 4.25 – 4.36 (br m, 3H), 5.07 (br s, 1H), 5.13 (br s, 1H). ^13^C NMR (101 MHz, CDCl_3_) δ 14.6, 23.7, 28.5, 33.3, 33.4, 40.4, 42.9, 52.5, 53.4, 57.9, 62.4, 68.5, 70.3, 70.4, 70.4, 79.3, 156.1, 166.0, 166.6, 170.5. HRMS (m/z): [M + H]^+^ calcd. for C_25_H_46_N_7_O_5_, 524.3555; found, 524.3511.

#### 4-(4-(6-Aminohexyl)Piperazin-1-yl)-*N*-Cyclopentyl-6-Ethoxy-1,3,5-Triazin-2-Amine (5, UOSD005)

**3** (3.0 mg, 6.1 μmol) was dissolved in CH_2_Cl_2_ (0.5 mL) and trifluoroacetic acid (0.25 mL) was added. After 1 h the solvents were evaporated and the product purified by semi-preparative RP-HPLC and then neutralised using Amberlyst A21 ion exchange resin to give **5** (2.3 mg, 5.9 μmol, 96% yield) as a waxy, white solid. HRMS (m/z): [M + H]^+^ calcd. for C_20_H_38_N_7_O, 392.3132; found, 392.3104. Analytical RP-HPLC *R*_t_ = 10.72 min, purity 100%.

#### 4-(4-(2-(2-(2-Aminoethoxy)Ethoxy)Ethyl)Piperazin-1-yl)-*N*-Cyclopentyl-6-Ethoxy-1,3,5-Triazin-2-Amine (6, UOSD009)

**4** (10.0 mg, 19.1 μmol) was reacted according to the procedure for **5** to give **6** (6.3 mg, 13.5 μmol, 78% yield) as a yellow oil. HRMS (m/z): [M + H]^+^ calcd. for C_20_H_38_N_7_O_3_, 424.3031; found, 424.3037. Analytical RP-HPLC *R*_t_ = 10.27 min, purity 96.2%.

*N*-(ad-amantan-1-yl)-4-ethoxy-6-(piperazin-1-yl)-1,3,5-triazi n-2-amine **7**, *N*-(Adamantan-1-yl)-4-ethoxy-6-(4-methylpipera zin-1-yl)-1,3,5-triazin-2-amine **15**, and *N*-(adamantan-1-yl)-4-et hoxy-6-(4-(2-fluoroethyl) piperazin-1-yl)-1,3,5-triazin-2-amine **16** were prepared as previously reported (Yrjölä et al., 2013, 2015). **7**, **15**, and **16** were characterised by NMR spectroscopy and HRMS (data not shown), and sample purity of test compounds was 97.2, 99.5, and 98.5%, respectively.

#### *tert*-Butyl *N*-(2-(4-(4-((Adamantan-1-yl)Amino)-6-Ethoxy-1,3,5-Triazin-2-yl)Piperazin-1-yl)Ethyl) Carbamate (8)

**7** (120 mg, 0.33 mmol) and 2-(Boc-amino) ethyl bromide (120.0 mg, 0.54 mmol) were reacted according to the procedure for **3** and purified by silica gel flash chromatography (5% MeOH/CH_2_Cl_2_) to give a ∼2:1 mixture of **8** and the Boc-deprotected analogue of **8** (25 mg). Since **8** was not analysed for biological activity, the ∼2:1 mixture was carried through to the Boc-deprotection reaction (**11**, described later) (with NMR data reported for **11**).

#### *tert*-Butyl *N*-(6(4-(4-(Adamantan-1-yl)Amino)-6-Ethoxy-1,3,5-Triazin-2-yl)Piperazin-1-yl)Hexyl) Carbamate (9, UOSD016)

**7** (120 mg, 0.33 mmol) and *tert*-butyl *N*-(6-bromohexyl) carbamate (90 mg, 0.32 mmol) were reacted according to the procedure for **3** and purified by silica gel flash chromatography (50–80% EtOAc/hexane) to give **9** (82 mg, 0.15 mmol, 44% yield) as a pale yellow oil. ^1^H NMR (400 MHz, CDCl_3_) δ 1.32 (m, 7H), 1.41 – 1.53 (m, 11H), 1.68 (m, 8H), 2.08 (m, 9H), 2.33 (t, *J* = 6.7 Hz, 2H), 2.43 (m, 4H), 3.10 (m, 2H), 3.79 (m, 4H), 4.28 (m, 2H), 4.51 (br s, 1H), 4.88 (br s, 1H). ^13^C NMR (CDCl_3_) δ 14.7, 26.8, 27.3, 28.6, 29.6, 30.1, 36.6, 36.7, 40.7, 42.0, 43.3, 51.5, 53.2, 58.8, 62.5, 79.1, 156.1, 161.0, 165.8, 170.5. HRMS (m/z): [M + H]^+^ calcd. for C_30_H_52_N_7_O_3_, 558.4126; found, 558.4107. Analytical RP-HPLC *R*_t_ = 18.96 min, purity 100%.

#### *tert*-Butyl *N*-(2-(2-(2-(4-(4-((Adamantan-1-yl)Amino)-6-Ethoxy-1,3,5-Triazin-2-yl)Piperazin-1-yl)Ethoxy) Ethoxy)Ethyl)Carbamate (10, UOSD015)

**7** (80.0 mg, 0.22 mmol) and *tert*-butyl *N*-{2-[2-(2-bromoethoxy) ethoxy]ethyl}carbamate (70.0 mg, 0.23 mmol) were reacted according to the procedure for **3** and purified by silica gel flash chromatography (5% MeOH/CH_2_Cl_2_) to give **10** (73 mg, 0.12 mmol, 55% yield) as a pale yellow oil. ^1^H NMR (400 MHz, CDCl_3_) δ 1.33, (m, 3H), 1.42 (s, 9H), 1.66 (m, 6H), 2.05 (br m, 9H), 2.59 (m, 4H), 2.68 (m, 2H), 3.29 (m, 2H), 3.51 (m, 2H), 3.59 (m, 4H), 3.67 (m, 2H), 3.84 (m, 4H), 4.26 (m, 2H), 4.92 (br s, 1H), 5.11 (br s, 1H). ^13^C NMR (101 MHz, CDCl_3_) δ 14.7, 28.5, 29.6, 36.6, 40.5, 41.9, 42.8, 51.6, 53.4, 57.8, 62.3, 68.3, 70.3, 70.4, 70.4, 79.3, 156.1, 165.7, 166.1, 170.4. HRMS (m/z): [M + H]^+^ calcd. for C_30_H_52_N_7_O_5_, 590.4024; found, 590.3974. Analytical RP-HPLC *R*_t_ = 18.69 min, purity 96.9%.

#### *N*-(Adamantan-1-yl)-4-(4-(2-Aminoethyl)Piperazin-1-yl)-6-Ethoxy-1,3,5-Triazin-2-Amine (11, UOSD007)

**8** (10 mg, 19.9 μmol) was reacted according to the procedure for **5** to give **11** as a white waxy solid (5.4 mg, 13.45 μmol, 67% yield). ^1^H NMR (400 MHz, CD_3_OD) δ 1.38 (t, *J* = 7.1 Hz, 3H), 1.74 (m, 6H), 2.10 (m, 3H), 2.14 (m, 6H), 2.69 (m, 4H), 2.76 (m, 2H), 3.14 (m, 2H), 3.94 (br m, 4H), 4.46 (m, 2H). HRMS (m/z): [M + H]^+^ calcd. for C_21_H_36_N_7_O, 402.2976; found, 402.2961. Analytical RP-HPLC *R*_t_ = 14.12 min, purity 97.1%.

#### *N*-(Adamantan-1-yl)-4-(4-(6-Aminohexyl)Piperazin-1-yl)-6-Ethoxy-1,3,5-Triazin-2-Amine (12, UOSD010)

**9** (13 mg, 23.3 μmol) was reacted according to the procedure for **5** to give **12** as a white waxy solid (9.2 mg, 20.1 μmol, 86% yield). HRMS (m/z): [M + H]^+^ calcd. for C_25_H_44_N_7_O, 458.3602; found, 458.3612. Analytical RP-HPLC *R*_t_ = 14.50 min, purity 100%.

#### *N*-(Adamantan-1-yl)-4-(4-(2-(2-(2-Aminoethoxy) Ethoxy)Ethyl)Piperazin-1-yl)-6-Ethoxy-1,3,5-Triazin-2-Amine (13, UOSD008)

**10** (10.0 mg, 17.0 μmol) was reacted according to the procedure for **5** to give **13** (8.0 mg, 16.3 μmol, 96% yield) as a white waxy solid. ^1^H NMR (400 MHz, CD_3_OD) δ 1.37, (t, *J* = 6.9 Hz, 3H), 1.74 (m, 6H), 2.10 (br m, 2H), 2.14 (br m, 7H), 3.12 (t, *J* = 5.1 Hz, 2H), 3.34 (m, 4H), 3.40 – 3.51 (br m, 6H), 3.71 (m, 6H), 3.87 (m, 2H), 4.44 (m, 2H). ^13^C NMR (101 MHz, CD_3_OD) δ 14.54, 30.94, 37.45, 40.55, 41.84, 42.40, 49.85, 52.80, 53.78, 57.54, 65.53, 67.86, 71.29, 71.35, 162.69, 163.05, 165.59. HRMS (m/z): [M + H]^+^ calcd. for C_25_H_44_N_7_O_3_, 490.3500; found, 490.3478. Analytical RP-HPLC *R*_t_ = 13.47 min, purity 100%.

#### *N*-(2-(2-(2-(4-(4-((Adamantan-1-yl)Amino)-6-Ethoxy-1,3,5-Triazin-2-yl)Piperazin-1-yl)Ethoxy)Ethoxy) Ethyl)Acetamide (14, UOSD017)

To a solution of **13** (10.0 mg, 20.4 μmol) in CH_2_Cl_2_ (1 mL) was added Et_3_N (8 μL, 61 μmol) and acetic anhydride (2 μL, 22 μmol). After 30 min, the solvents were removed and the product was purified by semi-preparative RP-HPLC to give **14** (4.0 mg, 7.5 μmol, 37% yield) as a white waxy solid. HRMS (m/z): [M + H]^+^ calcd. for C_27_H_46_N_7_O_4_, 532.3606; found, 532.3571. Analytical RP-HPLC *R*_t_ = 15.53 min, purity 100%.

### Cell Lines and Maintenance

Cell culture medium and reagents were purchased from Thermo Fisher Scientific (Waltam, MA, United States), and plasticware was purchased from Corning (Corning, NY, United States) unless otherwise noted. HEK Flp-in wild-type (wt) cells were purchased from Invitrogen (Carlsbad, CA, United States). HEK Flp-in hCB_2_ and wt lines were maintained in high glucose Dulbecco’s Modified Eagle’s Medium (DMEM, Thermo Fisher Scientific medium formulation #11995) supplemented with 10% foetal bovine serum (FBS, New Zealand-origin, Moregate Biotech, Brisbane, Australia) and appropriate selection antibiotics, and incubated in 5% CO_2_ at 37°C in a humidified atmosphere. The HEK Flp-in line stably expressing hCB_2_ with an amino-terminal haemagglutinin (HA) tag has been described previously (Soethoudt et al., 2017, see pERK assay).

### Radioligand Binding Assays

The hCB_2_-expressing HEK cell line used for binding assays was first reported in Grimsey et al. (2011), and the hCB_1_-expressing HEK cell line was first reported in Finlay et al. (2017). Cells were harvested and membranes prepared as described previously (Finlay et al., 2017) and subsequently homologous and heterologous competition assays were performed. [^3^H]-CP 55,940 (final concentration 0.5 nM for hCB_2_ and 0.75 nM for hCB_1_; PerkinElmer, Waltham, MA, United States), HEK membranes (2.25 μg per point for hCB_2_ and 2.5 μg per point for hCB_1_), concentration series of CP 55,940 (Tocris, Bristol, United Kingdom) for homologous competition assays to obtain K_d_ and concentration series or single concentrations of 2,4,6-trisubstituted 1,3,5-triazines for heterologous competition assays to obtain K_i_s at hCB_2_ or to determine binding extent at 10 μM at hCB_1_, respectively, were individually prepared in binding buffer (50 mM HEPES pH 7.4, 1 mM MgCl_2_, 1 mM CaCl_2_, 2 mg mL^-1^ low endotoxin bovine serum albumin; BSA, ICPBio, Auckland, New Zealand), and incubated together for 1 h at 30°C. A 96-well Harvest Plate (PerkinElmer) was pre-soaked with 0.1% wv^-1^ polyethylenimine (PEI; Sigma-Aldrich) for 1 h at room temperature, and at the end of the incubation placed on a vacuum manifold and washed with 200 μL ice-cold wash buffer (50 mM HEPES pH 7.4, 500 mM NaCl, 1 mg mL^-1^ BSA). Immediately following this, samples were transferred onto the harvest plate and subjected to a further three 200 μL washes with ice-cold wash buffer. Harvest plates were dried overnight, followed by the addition of 50 μL Irga-Safe Plus scintillation fluid (PerkinElmer) to each well. Controls to verify that radioligand depletion was less than 10% were also included. After a 30 min delay, plates were detected for 2 min per well in a MicroBeta^®^ TriLux (PerkinElmer). Homologous competition assays were utilised to determine K_d_ at hCB_2_ for CP 55,940 and conformed to homologous binding assumptions (Motulsky and Christopoulos, 2003). Both homologous and heterologous binding data were modelled utilising predefined equations in GraphPad Prism (v7.03; GraphPad Software Inc., La Jolla, CA, United States). In heterologous binding assays, curves were constrained for compounds that did not fully displace [^3^H]-CP 55,940, whereby the bottom of the curve was defined by maximum CP 55,940 displacement of [^3^H]-CP 55,940.

### Internalisation Assays

Internalisation assays were carried out by fluorescent immunocytochemistry with selective antibody labelling of cell surface receptors as described previously with minor modifications (Grimsey et al., 2008, 2011). In brief, HEK Flp-in hCB_2_ cells were seeded at 4.5 × 10^4^ cells/well in poly-D-lysine (Sigma-Aldrich) treated clear 96-well Nunc^TM^ plates (Thermo Fisher Scientific) 18–24 h prior to stimulation. All drugs and reagents for internalisation assays were prepared in high glucose DMEM supplemented with 1 mg mL^-1^ BSA (basal medium). Cells were equilibrated in basal medium for 30 min at 37°C, and incubated with anti-mouse monoclonal HA.11 (901503; BioLegend, San Diego, CA, United States) diluted 1:500 in basal medium for 30 min at room temperature (thereby restricting primary antibody labelling to cell surface hCB_2_). Cells were then briefly washed with basal medium and then incubated with vehicle or drug for 1 h at 37°C. At the conclusion of the drug incubation, plates were placed on ice for at least 2 min to halt membrane trafficking and briefly washed with room temperature basal medium. Alexa Fluor^®^ 488-conjugated goat anti-mouse secondary antibody (Invitrogen) diluted 1:300 in basal medium was then applied to cells and incubated for 30 min at room temperature (thereby restricting secondary antibody labelling to only residual primary antibody-bound hCB_2_ remaining on the cell surface at the end of drug treatment). Cells were then washed twice with basal medium and fixed in 4% paraformaldehyde for 10 min. Following two phosphate buffered saline (PBS) washes, cell nuclei were labelled with Hoechst 33258 (4 mg mL^-1^ in water; Sigma-Aldrich) diluted 1:500 in PBS with 0.2% Triton X-100.

Image acquisition and analysis were based on previously described methods (Grimsey et al., 2008; Finlay et al., 2016). Briefly, images were captured with the ImageXpress^®^ Micro XLS Widefield High-Content Analysis System (Molecular Devices, Sunnyvale, CA, United States) (10× objective, 4 sites per well), and receptor internalisation quantified using MetaXpress^®^ software (v6.2.3.733, Molecular Devices) by calculating the fluorescence intensity per cell above background. Data were normalised to vehicle-treated (100%).

### cAMP Assays

Cellular cAMP levels were measured using a real-time BRET biosensor (CAMYEL) as previously described (Cawston et al., 2013). In brief, cells were seeded in 10 cm dishes one day prior to transfection. The medium was then replaced and cells transfected with 5 μg of pcDNA3L-His-CAMYEL (ATCC, Manassas, VA, United States) using linear PEI (MW 25 kDa; Polysciences, Warrington, PA, United States) with a DNA:PEI ratio of 1:6. Approximately 24 h after transfection, cells were trypsinised and re-plated in poly-D-lysine treated white 96-well plates (Corning), at a density of 5–6 × 10^4^ cells per well. For experiments involving pertussis toxin (PTX; Sigma-Aldrich) pre-treatment, cells were seeded in half the usual volume of medium, and then ∼5 h later, 2× concentrated PTX or vehicle (50% glycerol, 50 nM Tris–HCl pH 7.5, 10 mM glycine, 500 mM NaCl) were prepared in medium and added to the existing plating medium (giving rise to a final PTX concentration of 100 ng mL^-1^). Approximately 24 h after plating (or 16–20 h after applying PTX/vehicle in experiments involving PTX treatment), cells were washed once with Hank’s Balanced Salt Solution (HBSS, Thermo Fisher Scientific, catalogue #14025-134), and then equilibrated for 30 min in HBSS supplemented with 1 mg mL^-1^ BSA. Coelenterazine h (final concentration 5 μM; NanoLight Technologies, Pinetop, AZ, United States) was added to each well and incubated for 5 min. Forskolin was added (final concentration 5 μM) and incubated for 6 min (allowing enough time for the induced cAMP increase to plateau), prior to vehicle/drug addition. All drugs and reagents were prepared in HBSS supplemented with 1 mg mL^-1^ BSA. SR 144528 was a generous gift from Roche (Basel, Switzerland).

Assays were carried out in technical duplicate. Emissions were detected at 460 nm (Rluc) and 535 nm (YFP) with either a LUMIstar^®^ Omega luminometer (BMG Labtech, Ortenberg, Germany) or CLARIOstar^®^ (BMG Labtech). BRET ratios (535 nm emissions/460 nm emissions, where an increase in ratio corresponds to a decrease in cAMP) were normalised to pre-forskolin reads (i.e., with coelenterazine h present) to remove any variability present prior to addition of forskolin and drug/vehicle. A Lowess curve (GraphPad Prism) was fitted to individual technical replicates within an assay so that cAMP signalling data could be extrapolated from any time point as opposed to only those that were empirically measured (typically every 30–60 s). Data were normalised to matched vehicle (0%) and forskolin (100%) treatments, allowing compilation of data from independent experiments.

### pERK Assays

ERK phosphorylation assays were performed on HEK Flp-in hCB_2_ and HEK Flp-in wt cell lines. Cells were seeded at a density of 4.5 × 10^4^ cells per well in poly-D-lysine treated clear 96-well Nunc^TM^ plates; for PTX experiments cells were plated in the presence of PTX (100 ng mL^-1^) or vehicle. 24–25 h later, medium was removed and replaced with basal medium (DMEM supplemented with 1 mg mL^-1^ BSA). Cells were equilibrated for 3 h at 37°C followed by drug stimulations carried out at 37°C for 4 min. All drugs were prepared in basal medium at 2× final concentration. An inhibitor of ERK phosphorylation, U0126 (Cell Signaling Technology, MA, United States) at 10 μM, and a pERK pathway stimulant, phorbol 12-myristate 13-acetate (PMA; Sigma-Aldrich) at 100 nM, both incubated for 15 min, were utilised as reference points. At the conclusion of the drug incubation, plates were placed on ice, and immediately lysed by adding 20 μL of ice-cold lysis buffer (from AlphaLISA^®^ Kit; details follow). Detection was performed using the AlphaLISA^®^ SureFire^®^ Ultra^TM^ p-ERK 1/2 (Thr202/Tyr204) Assay Kit (PerkinElmer), according to manufacturer instructions, and plates read in a CLARIOstar^®^ reader (BMG Labtech) using standard AlphaScreen-compatible filters. Data were normalised to matched U0126 (0%) and PMA (100%) treatments, allowing compilation of data from independent experiments.

### General Analysis and Statistics

All sigmoidal concentration-response curves were obtained by fitting three-parameter (Hill slope constrained to 1) nonlinear regression curves (GraphPad Prism). Other than for bias analysis (see section “Bias Analysis”), statistical analyses were performed on the means from three to four independent experiments using Sigmaplot^TM^ v13.0 (Systat Software Inc., San Jose, CA, United States). The Shapiro–Wilk test for normality and Brown–Forsythe test for equal variance were performed to verify the datasets were appropriate for analysis with parametric statistical tests; all sets obtained a pass result of *p* > 0.05. A student’s *t*-test, one-way ANOVA, or two-way ANOVA was carried out as appropriate for the number of conditions and factors under comparison, and if a statistically significant difference (*p* < 0.05) was detected, the Holm–Šídák (Shaffer, 1986) *post hoc* test was used to test for significant differences within/between groups.

### Bias Analysis

Data from independent internalisation, cAMP and pERK experiments were normalised to the vehicle response (0%) and the mean maximum response measured for any ligand in that assay (100%) so that relative efficacies could be assessed on the same scale. Bias analysis was performed nearly exactly as described by van der Westhuizen et al. (2014) in GraphPad Prism. The derived parameters relating to bias analysis are explained in the results, section 3.6. Methodology, including specific equations for fitting the operational model and subsequently calculating ΔlogR and ΔΔlogR with associated standard errors, were equivalent to this prior publication except that we opted not to subjectively classify ligands into full versus partial agonists (as was done in van der Westhuizen et al., 2014), instead treating all ligands other than the reference as potentially partial (and therefore having the general operational model fitted for all ligands, rather than assuming equivalent efficacy for all qualitatively full agonists). This approach was validated by Zhu et al. (2018), and indeed made no meaningful difference to our own findings when empirically tested. The maximal system efficacy, basal response and “*n*” (transducer function slope) were set to be “shared for all datasets” as per the default constraints suggested by van der Westhuizen et al. (2014). The mean (and associated error) of all independent CP 55,940 experiments was utilised as the reference condition for comparison with all ligands’ independent experiment data analysed separately, including the independent CP 55,940 experiment data which was re-analysed with reference to the mean data in order to obtain an indication of error for this ligand. Tests for statistical significance of differences between ΔΔlogR values were carried out in GraphPad Prism utilising two-way ANOVA with Holm–Šídák *post hoc* multiple comparisons test. *p*-values are indicated graphically as: ^∗^ <0.05, ^∗∗^ <0.005, and ^∗∗∗^ <0.001.

## Results

### Chemical Synthesis

**SCHEME 1:**
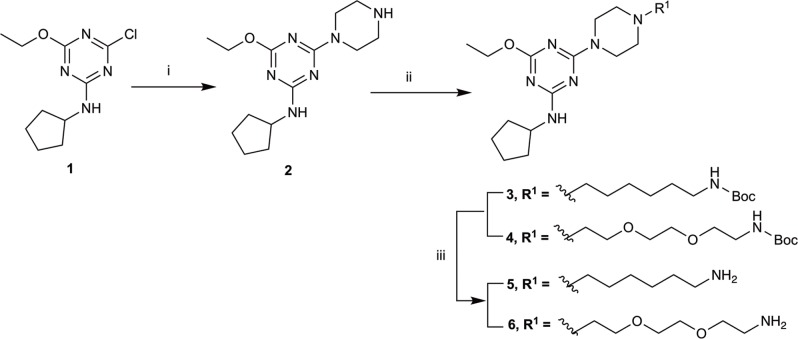
Synthesis of *N*-cyclopentyl series. (i) piperazine, *N*,*N*-diisopropylethylamine, THF, reflux, 12 h, 48%; (ii) *tert*-butyl *N*-(6-bromohexyl) carbamate or *tert*-butyl *N*-{2-[2-(2-bromoethoxy)ethoxy]ethyl}carbamate, K_2_CO_3_, NaI, dioxane, reflux, 24 h, 66–72%; and (iii) CH_2_Cl_2_, trifluoroacetic acid, 78–96%.

**SCHEME 2:**
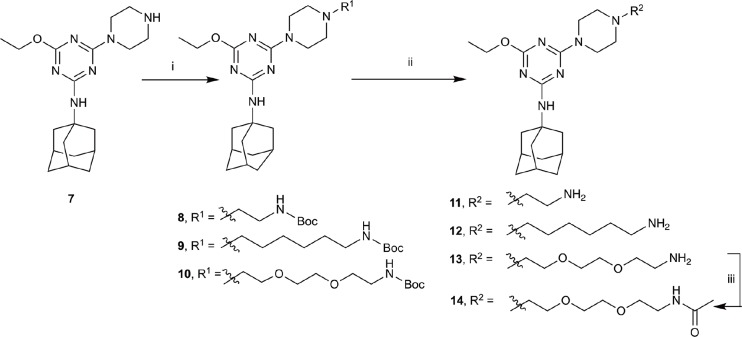
Synthesis of *N*-adamantanyl series. (i) *tert*-butyl *N*-(6-bromohexyl)carbamate or *tert*-butyl *N*-{2-[2-(2-bromoethoxy)ethoxy]ethyl}carbamate or 2-(Boc-amino)ethyl bromide, K_2_CO_3_, NaI, dioxane, reflux, 24 h, 44–55%; (ii) CH_2_Cl_2_, trifluoroacetic acid, 67–96%; and (iii) CH_2_Cl_2_, Et_3_N, acetic anhydride, 30 min, 37%.

The piperazine-containing 1,3,5-triazines **1** and **7**, assembled following literature protocols (compound “26a” in Yrjölä et al., 2013; compound “8” in Yrjölä et al., 2015, respectively), were alkylated with various alkyl bromide linkers containing a Boc-protected amine at the terminus to give **3**, **4**, **8**, **9** and **10** (Schemes 1, 2). These were then Boc-deprotected using acidic conditions to give primary amines **5**, **6**, **11**, **12**, and **13**. One example of an acetylated linker (**14**) was also synthesised. Thirteen compounds were biologically tested (Table 1); five compounds had been reported previously (**1**, **2**, **7**, **15**, **16**) (Yrjölä et al., 2013, 2015), and the remainder were novel (**5**, **6**, **9**–**14**). The clogP and clogD_7.4_ of the compounds are shown in Table 1. Compounds were solubilised in dimethylsulfoxide (DMSO) to produce 31.6 mM stock solutions for pharmacological assays, other than **7** which exhibited a lower solubility in DMSO (to 1 mM). **7** was therefore assayed in a higher final DMSO content (1%) than the other compounds (0.1%). Both vehicle concentrations were controlled for in all assays (negative/vehicle and positive controls).

### Binding Affinity

CP 55,940, a well characterised synthetic cannabinoid, was utilised in tritiated form as the radioligand to assess orthosteric binding of the compounds of interest to hCB_2_. [^3^H]-CP 55,940 was measured to have a pK_d_ of 8.89 (± 0.07) at hCB_2_, which is similar to that previously published (Soethoudt et al., 2017).

Compounds **1**, **2**, **5**–**7**, and **7**–**16** were subjected to a 10 μM heterologous competition binding screen, subsequent to which those compounds which displaced more than 60% of the competing radioligand were advanced to determine binding affinity (Table 1). All cyclopentyl substituted triazines (**1**, **2**, **5**, and **6**) failed to displace, or barely displaced, [^3^H]-CP 55,940. The three adamantanyl substituted triazines with an alkyl chain, aminohexyl-piperazinyl-**12**, the corresponding Boc-protected analogue **9**, and the short chain aminoethyl–piperazinyl derivative **11** were moderate binders which approached but did not exceed our displacement cut-off and so K_i_ values were not determined.

The remaining six compounds displaced more than 60% of the competing radioligand and were therefore advanced in our study to determine binding affinity (K_i_) and later characterise functionally. Of these, **16** and **15** had the highest affinities with pK_i_s of 8.79 (± 0.03) and 8.37 (± 0.18), respectively. A ∼100-fold reduction in hCB_2_ affinity was measured for piperazinyl-**7** (pK_i_ 6.38 ± 0.16) compared to methylpiperazinyl-**15**, despite the difference of just a methyl group between the two analogues. The triazines containing a 1,2-diethoxyethane (PEG2)-piperazinyl moiety, amino-PEG2-piperazinyl-**13** and the corresponding Boc-protected (**10**) and acetylated (**14**) analogues, exhibited measurable binding, however introduction of this PEG2 linker reduced binding affinity by approximately 2–3 log units compared to methylpiperazinyl-**15**. Functional characterisation at hCB_2_ was therefore undertaken for **7**, **10**, **13**, **14**, **15**, and **16**, as well as CP 55,940 which we utilised as a reference ligand as this was expected to be a full agonist at hCB_2_ in every pathway we intended to evaluate (Soethoudt et al., 2017).

We were also interested to determine whether these six compounds exhibited any CB_1_ orthosteric binding. A 10 μM heterologous competition radioligand binding screen revealed that only **15** and **16** displaced more than 60% of [^3^H]-CP 55,940 (0.75 nM), however neither compound produced full displacement at this concentration (Table 1).

### Internalisation

**FIGURE 1:**
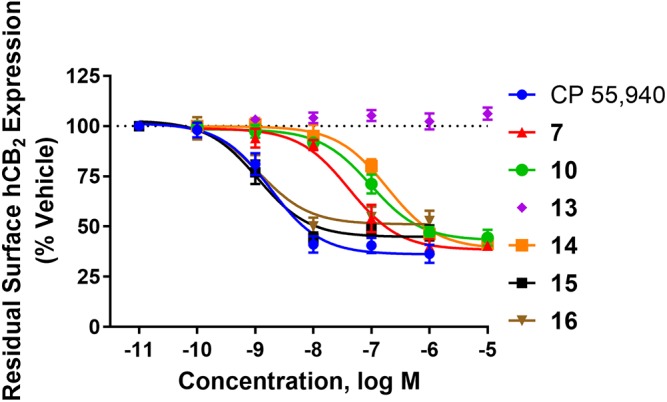
Internalisation of hCB_2_ in response to CP 55,940 and 2,4,6-trisubstituted 1,3,5-triazines. Internalisation of hCB_2_ in HEK Flp-in hCB_2_ cells in response to a 1 h stimulation with varying concentrations of CP 55,940 or test compounds. Data are presented as mean ± SEM from 3–4 independent experiments, and were normalised to vehicle-treated (100%).

hCB_2_ internalisation was assessed in response to a 1 h stimulation with **7**, **10**, **13**, **14**, **15**, **16**, and CP 55,940. All compounds except **13** behaved as agonists in this pathway and this agonist-induced internalisation was concentration-dependent (Figure 1 and Table 2). CP 55,940 internalised ∼66% of starting surface hCB_2_, which was a very similar response to that observed previously stimulating CB_2_ with HU-308 (Grimsey et al., 2011). Efficacies were comparable between CP 55,940 and all compounds that induced receptor internalisation, except for **16** which was significantly less efficacious (*p* < 0.001, one-way ANOVA). The rank order of potencies generally correlated with the rank order of binding affinity, with the exception that **15** had an equivalent internalisation potency as CP 55,940 despite having approximately half a log unit lower affinity. **13** appears to be a neutral antagonist in this pathway at the time point assayed as it was not significantly different from the vehicle-treated condition (*p* = 0.111 at 10 μM, *t*-test). As there was no measurable efficacy for this compound, an EC_50_ could not be determined.

**Table 2:** Summary data for hCB_2_ functional assays carried out on CP 55,940 and 2,4,6-trisubstituted 1,3,5-triazines.

	Internalisation (1 h)			cAMP (5 min)			pERK (4 min)		
Compound #	pEC_50_ (±SEM)	*E*_max_ (±SEM)^a^	*n*	pEC_50_ (±SEM)	*E*_max_ (±SEM)^b^	*n*	pEC_50_ (±SEM)	*E*_max_ (±SEM)^c^	*n*
CP 55,940	8.72 (0.09)	–65.1 (2.4)	4	8.48 (0.01)	–41.7 (1.0)	3	8.03 (0.06)	+10.29 (0.94)	3
**7**	7.34 (0.15)	–61.4 (2.7)	4	7.07 (0.15)	–43.0 (3.0)	3	5.74 (0.05)	+2.06 (0.08)	3
**10**	7.05 (0.13)	–57.4 (2.3)	4	6.98 (0.11)^Δ^	–33.0 (0.9)^Δ^	3	5.97 (0.04)	+10.18 (1.16)	3
**13**	Not Measurable	+6.2 (3.1)^∗^	3	6.10 (0.17)	+44.7 (4.6)	3	6.27 (0.07)	+1.22 (0.13)	3
**14**	6.76 (0.11)	–61.8 (1.6)	4	6.02 (0.17)^Δ^	–29.8 (0.5)^Δ^	3	5.13 (0.06)^∧^	+4.66 (0.16)^∧^	3
**15**	8.91 (0.11)	–56.5 (1.5)	4	8.10 (0.11)	–39.6 (1.2)	3	7.45 (0.07)	+9.96 (0.53)	3
**16**	8.92 (0.09)	–50.0 (2.7)	4	8.55 (0.16)	–35.6 (2.2)	3	7.91 (0.08)	+7.63 (0.25)	3


*^*a*^Internalisation *E*_*max*_ represented as reduction (-) or increase (+) in surface expression, as a percentage of vehicle-treated control. ^*b*^cAMP *E*_*max*_ represented as reduction (-) or increase (+) in cAMP as a percentage of forskolin-treated (100%) minus Vehicle-treated (0%) controls. ^*c*^pERK *E*_*max*_ indicates increase in pERK as a percentage of PMA (100%) and U0126 (0%) controls, less Vehicle-treated control. ^∗^A concentration-response curve could not be fitted, therefore efficacy at the highest concentration tested (i.e., 10 μM) is noted instead of *E*_*max*_. ^Δ^Parameters are approximate due to the possible influence of non-CB_*2*_-mediated effects. ^∧^A fully defined concentration-response curve could not be obtained, therefore this pEC_*50*_ can only be considered approximate, and efficacy at the highest concentration tested (i.e., 10 μM) is noted instead of *E*_*max*_. Column “*n*” indicates the number of independent experiments performed.*

### Cyclic AMP (cAMP)

**FIGURE 2:**
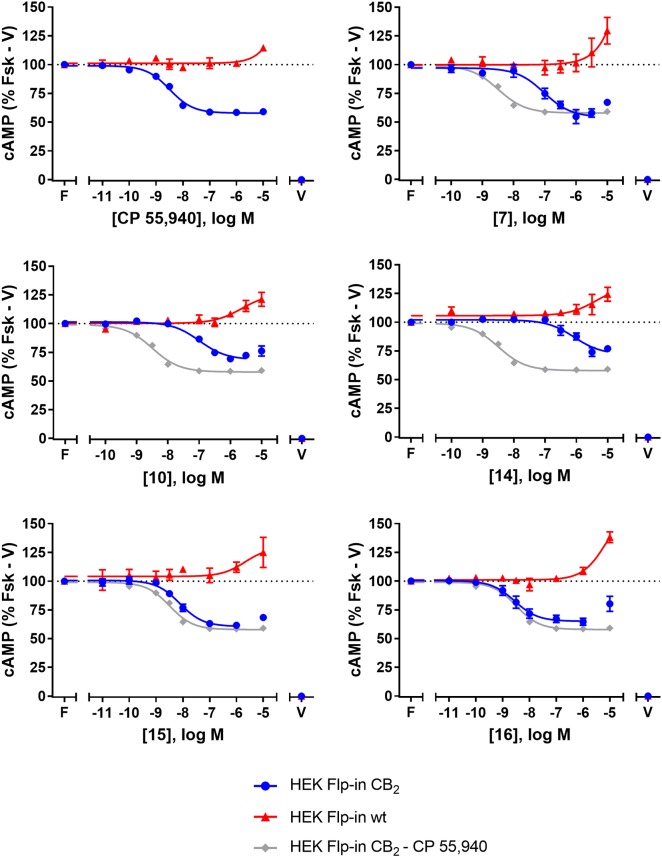
cAMP signalling in HEK Flp-in hCB_2_ and HEK Flp-in wt cells in response to CP 55,940 and 2,4,6-trisubstituted 1,3,5-triazines. Concentration-response curves for modulation of cAMP concentration in HEK Flp-in hCB_2_ and HEK Flp-in wt cells (showing both hCB_2_- and non-hCB_2_-mediated effects) on stimulation with 5 μM forskolin and CP 55,940 or test compounds (other than **13**). Concentration-response curves were generated by plotting measurements at 5 min post-drug addition. These data were then normalised to vehicle-treated (V; 0%) and forskolin alone (F, Fsk; 100%). Data are presented as mean ± SEM from three independent experiments.

The effects of **7**, **10**, **13**, **14**, **15**, **16**, and CP 55,940 on forskolin-stimulated cAMP concentration were then investigated using a real-time CAMYEL cAMP biosensor assay (Jiang et al., 2007). Each compound, excluding **13**, inhibited cAMP production (as expected for CB_2_ agonists) and reached maximum efficacy (*E*_max_) ∼5 min after drug addition. Concentration-response curves for each compound were accordingly generated at 5 min post-drug addition (Figure 2). The remainder of the time course observed (up to 30 min) were unremarkable, with responses exhibiting a partial recovery towards forskolin alone. As shown in Table 2, *E*_max_ for all the compounds tested were similar to that of CP 55,940, with the exception of **10** and **14** which appeared to act as partial agonists (*p* = 0.049 and *p* = 0.004, respectively, one-way ANOVA).

To identify whether there were any non-hCB_2_-mediated effects on cAMP signalling, the HEK Flp-in wt line was assayed in the same manner. Application of **7**, **10**, **14**, **15**, **16**, and CP 55,940 all had an effect on cAMP signalling in the HEK Flp-in wt cells, in the form of an increase in cAMP at high concentration(s) (Figure 2). At the highest concentration tested, non-hCB_2_-mediated effects ranged from approximately 10 to 40% above forskolin. CP 55,940 had the least pronounced non-hCB_2_ signalling profile (low potency and low efficacy), while **16** and **7** had the most pronounced effects. Importantly, however, the majority of compounds reached maximal apparent hCB_2_-mediated efficacy at a lower concentration than non-hCB_2_-mediated effects were first detected. The exceptions were **14** and **10**, wherein the non-hCB_2_-mediated effects were only ∼5- to ∼23-fold (respectively) less potent than the cAMP inhibition induced in hCB_2_-expressing cells. These non-hCB_2_-mediated increases in cAMP likely counteracted the hCB_2_-induced suppression of cAMP production, thereby blunting the apparent *E*_max_ of these two compounds. We therefore do not feel it is possible to conclusively determine the CB_2_-mediated potency or efficacy for **10** or **14** in this assay.

**FIGURE 3:**
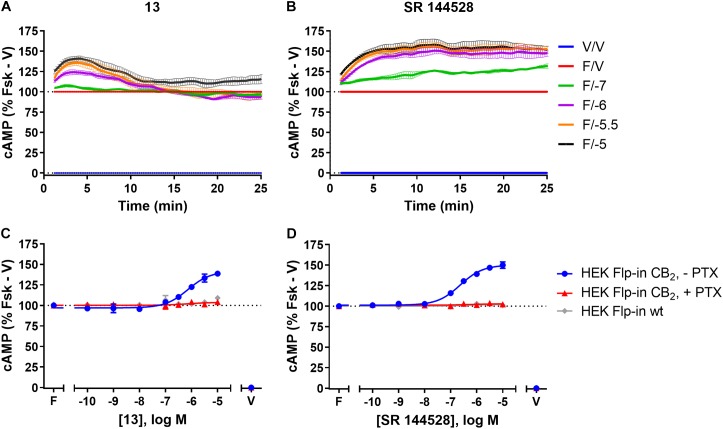
cAMP signalling in HEK Flp-in hCB_2_ and HEK Flp-in wt cells in response to **13** and prototypic inverse agonist, SR 144528. **(A,B)** Time courses for modulation of cAMP concentration by **13** **(A)** and SR 144528 **(B)** at active concentrations (noted in log units, in the presence of 5 μM forskolin “F/_”) on HEK Flp-in hCB_2_ cells. Data were normalised to vehicle-treated (“V/V”; 0%) and forskolin alone (“F/V”; 100%). **(C,D)** Concentration-response curves for modulation of cAMP concentration by compound **13** **(C)** and SR 144528 **(D)** (both in the presence of 5 μM forskolin) in HEK Flp-in hCB_2_ cells following 16–20 h pre-treatment in the absence or presence of PTX, or in HEK Flp-in wt cells. Concentration-response curves were generated by plotting each concentration measured at 5 min post-drug addition. These data were then normalised to vehicle-treated (V; 0%) and forskolin alone (F, Fsk; 100%). Data in all panels are presented as mean ± SEM from three independent experiments.

Amino-PEG2-piperazinyl-**13** did not inhibit forskolin-stimulated cAMP, but instead induced a transient concentration-dependent hCB_2_-mediated increase in cAMP, with a peak response time ∼4 min after addition, and reaching a *E*_max_ of ∼45% above forskolin at the highest concentration assayed (Figures 3A,C and Table 2). To assess whether this compound was acting as an inverse agonist via Gα_i_ or was stimulating cAMP production via a non-Gα_i_ pathway, cells were pre-treated with PTX to irreversibly inactivate Gα_i_ protein. As seen in Figure 3C, the observed cAMP signal for **13** was completely PTX-sensitive and thus Gα_i_-mediated, suggestive of inverse agonism. SR 144528, a CB_2_ inverse agonist (Portier et al., 1999), was therefore assayed for comparison with **13**. Stimulation with SR 144528 also resulted in a concentration-dependent increase in cAMP (Figure 3D), however an interesting point of difference between **13** and SR 144528 lies in their kinetics. As seen in Figure 3B, SR 144528 reached its *E*_max_ approximately 6 min after drug addition and this was maintained over the following 20 min, whereas compound **13** had a more rapid onset and returned to the cAMP concentration induced by forskolin alone within 10–15 min. Concentration response curves were plotted at 5 min for each compound. In this analysis, SR 144528 inhibited constitutive Gα_i_ activity with a greater efficacy than **13**, reaching a maximum of 55.4% (± 6.89) above forskolin (vs. 44.7% ± 4.6 for **13**). The increase by SR 144528 was confirmed to be PTX-sensitive (Figure 3D). Both of these compounds were also assayed in the HEK Flp-in wt line, and as seen in Figures 3C,D, did not exhibit any measurable non-hCB_2_-mediated effects.

### Phospho-ERK (pERK)

**FIGURE 4:**
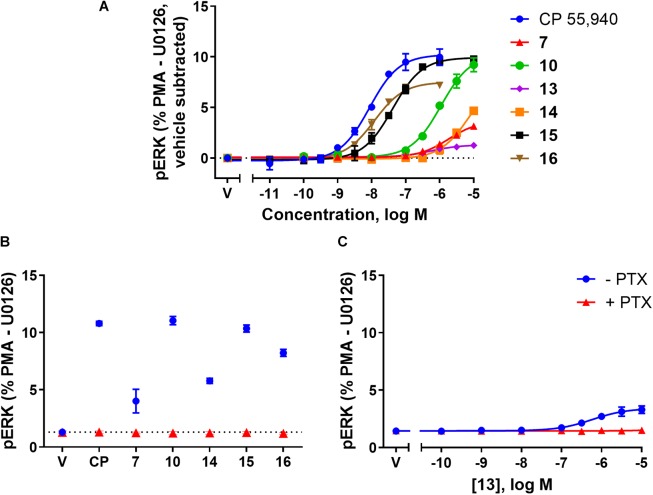
pERK activation via hCB_2_ in response to CP 55,940 and 2,4,6-trisubstituted 1,3,5-triazines. ERK1/2 phosphorylation (pERK) at 4 min post-drug addition in HEK Flp-in hCB_2_ cells. **(A)** Concentration-response curves for CP 55,940 and all test compounds. (V, vehicle) **(B)** Response at a single concentration (1 μM for CP 55,940 and **16**, 10 μM for others) following 27–28 h pre-treatment in the absence or presence of PTX (CP, CP 55,940). Dotted line indicates average vehicle (V) response. **(C)** Concentration-response curve for **13** following 27–28 h pre-treatment in the absence or presence of PTX. Data in all panels are presented as mean ± SEM from three independent experiments, and normalised to U0126 (0%) and PMA (100%). In panel **(A)** the vehicle response in each experiment was then subtracted such that the vehicle condition is represented as 0%.

The final functional pathway studied was the phosphorylation (activation) of ERK1/2. A time course experiment was first carried out for **7**, **10**, **13**, **14**, **15**, **16**, and CP 55,940 which revealed transient responses that reached maxima at 4 min for all compounds (data not shown). As such, concentration responses were carried out at 4 min. Efficacies are presented as percentage response above vehicle, relative to PMA as a positive control (being a strong activator of pERK, Besson et al., 2001) and U0126 as a negative control (being a MEK/ERK pathway blocker, Favata et al., 1998). At 4 min post-drug stimulation, CP 55,940 activated pERK with an *E*_max_ of 10.29% (± 0.94) (Figure 4A and Table 2). Compounds **10** and **15** had equivalent efficacies to CP 55,940. **16** activated pERK with an *E*_max_ of 7.63% (± 0.25), trending towards partial agonism, though this efficacy was not significantly different from CP 55,940 (*p* = 0.053, one-way ANOVA) (Figure 4A and Table 2). As shown in Table 2 and graphically represented in Figure 4A, **13** and **7**, are weak partial agonists, activating pERK with mean *E*_max_ of 1.22% (± 0.13) and 2.06% (± 0.08) respectively; these were significantly different from that of CP 55,940 (*p* < 0.001, one-way ANOVA) and vehicle (*p* = 0.004 and *p* < 0.001, respectively, paired *t*-test). These assays were also carried out in HEK Flp-in wt cells at each compound’s highest concentration tested to identify whether any non-specific effects on pERK activation were present; none were detected (data not shown).

To verify whether observed agonist activity was Gα_i_-mediated, pERK activation was measured in response to a maximally efficacious concentration of each ligand with and without PTX pre-treatment. As compound **13** appeared to be an antagonist/inverse agonist in the other functional assays carried out and its efficacy in this pERK pathway was extremely low, a full concentration response was carried out in the presence of PTX for this compound. Figures 4B,C demonstrate that all the ligands’ pERK responses were completely PTX-sensitive, confirming that all compounds are agonists in this pathway acting via Gα_i_.

### Comparison of Ligand Efficacy and Potency, and Bias Analysis

Having measured hCB_2_ binding affinities and responses in three functional assays, we were interested to compare the patterns of efficacy and potency between the 2,4,6-trisubstituted 1,3,5-triazine ligands and a prototypic CB_2_ ligand (CP 55,940), and determine whether any ligands exhibited evidence of bias between activation of these signalling pathways. As **13** was only an agonist in one pathway formal pathway bias calculations for this compound are impossible.

As an initial means of summarising and exploring our data, we compared concentration response *E*_max_ values (relative to the maximum measured response at any single concentration for each assay), and pEC_50_ values having subtracted each ligand’s own measured equilibrium binding affinity (pK_d_ or pK_i_ as applicable).

**FIGURE 5:**
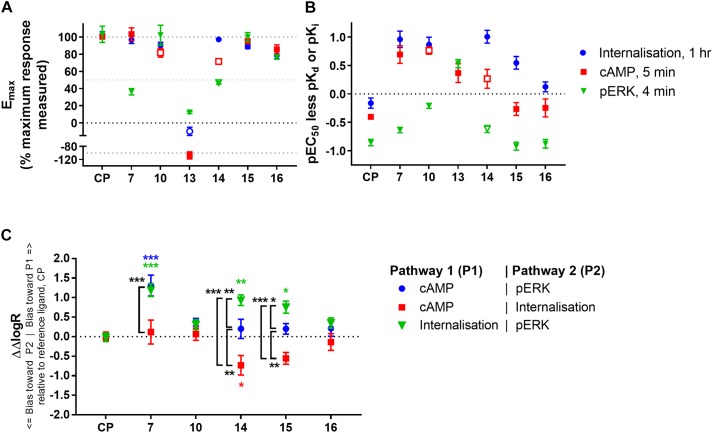
Comparison of ligand efficacy, potency (relative to binding affinity), and signalling pathway bias for CP 55,940 and 2,4,6-trisubstituted 1,3,5-triazines. **(A)** Internalisation, inhibition of cAMP production and stimulation of pERK *E*_max_ for each compound normalised to the overall maximum response measured at a single concentration in each pathway. Hollow symbols represent efficacy measures where conclusive *E*_max_ values could not be determined; for **10** and **14** cAMP *E*_max_ is approximate due to the presence of non-hCB_2_-mediated effects, for **14** pERK and **13** internalisation efficacy at 10 μM is plotted because fully-defined concentration-response curves could not be drawn. (CP, CP 55,940) **(B)** pEC_50_ for the three signalling pathways indicated less pK_d_ (for CP 55,940, CP) or pK_i_ (for the 2,4,6-trisubstituted 1,3,5-triazines). Hollow symbols represent potency measures where conclusive EC_50_ values could not be determined; for **10** and **14** cAMP EC_50_ is approximate due to the presence of non-hCB_2_-mediated effects, for **14** pERK EC_50_ is approximate due to its low potency. **(C)** Between-signalling pathway bias as represented by ΔΔlogR in comparison with CP 55,940 (CP) as a reference ligand. **13** could not be included in the bias quantitation as it is an agonist in only one pathway. The symbol “^∗^” in colour indicates significant difference from CP 55,940 for the same bias comparison; The symbol “^∗^” in black and white indicates significant difference for the comparison indicated by the associated bracket; significance levels as defined in the methods. Data in all panels are presented as mean ± SEM from three independent experiments.

Firstly inspecting efficacy (Figure 5A), CP 55,940 and **15** exhibited essentially equivalent efficacy in all three pathways measured, acting as full agonists relative to the series of compounds tested and in the cell model utilised. **16** also produced an equivalent response between all three assays, acting as a partial agonist in all pathways (inducing approximately 75–80% of maximal efficacy), though as per earlier analysis this was only found to be statistically different from CP 55,940 in the internalisation pathway. **10** appeared to be a full agonist in both the internalisation and pERK pathways. Although slightly lesser efficacy in the cAMP assay was measured for this compound, this may have been due to the presence of non-specific effects at active concentrations [see also the section “Cyclic AMP (cAMP)”]. The efficacy profile of **14** is difficult to judge due to the difficulty in estimating cAMP efficacy (due to non-CB_2_ mediated effects), and its low potency in the pERK assay preventing reliable determination of *E*_max_. Very interestingly, **7** acted as a full agonist in both the internalisation and cAMP assays, however was a weak partial agonist in the pERK assay with only ∼36% response relative to the maximal measured response across all ligands. Given that the rank order of efficacy for **16** versus **7** is different between the pERK pathway and the other two pathways studied (cAMP and Internalisation), this is an initial indication that there may be genuine bias away from pERK activation for **7** (e.g., Berg et al., 1998; Charfi et al., 2015).

In examining signalling assay potencies (EC_50_) relative to equilibrium binding affinity (Figure 5B), we first noted that CP 55,940 had the smallest EC_50_ range between all pathways. This, combined with its full efficacy in all pathways, supports its selection as a reference ligand for subsequent bias analysis (van der Westhuizen et al., 2014). CP 55,940’s potency for inducing internalisation was similar to its binding affinity (*p* = 0.127, one-way ANOVA), and the potency for eliciting a cAMP response was moderately (∼0.4 log units) lower than binding affinity (*p* = 0.010, one-way ANOVA). In contrast, the pERK response had a nearly 10-fold lower potency than affinity (*p* < 0.001, one-way ANOVA) and approximately 0.5 log unit lower potency than that for cAMP (*p* = 0.007, one-way ANOVA). This general relationship between cAMP and pERK potencies was consistent for all the 2,4,6-trisubstituted 1,3,5-triazines tested (other than **13**). **16** exhibited a similar pattern of EC_50_ relative to binding affinity to CP 55,940, however other compounds deviated from this pattern.

The EC_50_ pattern of **15** was also similar to CP 55,940 for cAMP and pERK, however its propensity to induce internalisation (relative to its equilibrium binding affinity) was approximately half a log unit more potent than would be predicted based on CP 55,940 (*p* < 0.001; two-way ANOVA). The pattern for **14** was similar to **15**, though it must be noted that due to its low potency we consider the cAMP and pERK parameters for this compound to be approximate. Both of these ligands therefore demonstrate some informal indication of bias towards induction of hCB_2_ internalisation. **7** and **10** also had seemingly high internalisation potencies relative to binding affinity (*p* < 0.001, two-way ANOVA), however these also exhibited greater potencies for inhibiting cAMP synthesis than would have been predicted from CP 55,940 (*p* < 0.001, two-way ANOVA), and **10** additionally induced ERK phosphorylation with a greater relative potency in comparison with CP 55,940 (*p* = 0.003, two-way ANOVA). Therefore, given that the relationship between the signalling pathway potencies (relative to binding affinity) is similar for **10** and CP 55,940 (i.e., no bias between signalling pathways is necessarily indicated), but the potencies for **10** are all greater than expected in comparison to CP 55,940, a high intrinsic coupling efficiency for activating CB_2_ is indicated for **10**. That is, half-maximal efficacy might be reached at a lower hCB_2_ occupancy with **10** than would be required for half-maximal efficacy when stimulating with CP 55,940. **7** seemed to act similarly to **10** in terms of having apparently high coupling efficiency for activating both internalisation and cAMP (in comparison with CP 55,940), however this was less apparent for pERK.

Given the noted indications of possible bias, we proceeded to carry out formal quantitative bias analysis utilising CP 55,940 as the reference ligand (Figure 5C and Table 3). We followed the method of van der Westhuizen et al. (2014), which is based on the seminal Black and Leff operational model (Black and Leff, 1983). This approach facilitates the calculation of a “transduction coefficient” (*τ*/K_A_) which is the coupling efficiency of an agonist to a signalling pathway (*τ*) relative to binding affinity (K_A_). This is calculated for each ligand in each signalling pathway and typically represented as a decimal logarithm (here referred to as logR). When considered relative to a reference ligand (ΔlogR) and compared between signalling pathways (ΔΔlogR), a measure of relative bias for activating one pathway over another is obtained. This parameter, and derived bias factor (BF, 10^ΔΔlogR^) which indicates fold differences in pathway activation bias, takes both potency and efficacy into account, removes system-specific factors (such as receptor expression level and capacity for signalling pathway activation), and allows for the possibility that receptor occupancy at the time point of the assay will not necessarily be the same as at equilibrium (i.e., K_A_ is not necessarily identical to K_d_ or K_i_). “*n*” is the transducer function slope, which links agonist concentration with the measured response.

**Table 3:** Signalling pathway transduction ratios (logR) and transducer function slopes (“*n*”), with between-pathway delta–delta transduction ratios (ΔΔlogR) and bias factors relative to reference ligand CP 55,940.

	logR [log(τ /K_A_)] (±SEM)			cAMP – pERK		cAMP – internalisation		Internalisation – pERK	
Compound #	Internalisation	cAMP	pERK	ΔΔlogR (±SEM)	Bias factor	ΔΔlogR (±SEM)	Bias factor	ΔΔlogR (±SEM)	Bias factor
CP 55,940	8.76 (0.1)	8.52 (0.02)	8.05 (0.03)	0.00 (0.04)	1.0	0.01 (0.10)	1.0	–0.02 (0.11)	1.0
**7**	7.42 (0.26)	7.29 (0.26)	5.51 (0.05)	1.31 (0.27)***	20.3	0.12 (0.31)	1.3	1.19 (0.16)***	15.4
**10**	7.09 (0.10)	6.90 (0.10)	6.06 (0.02)	0.37 (0.10)	2.4	0.07 (0.16)	1.2	0.31 (0.13)	2.0
**14**	6.81 (0.23)	5.83 (0.23)	5.16 (0.09)	0.20 (0.25)	1.6	–0.73 (0.25)*	0.2	0.93 (0.13)**	8.6
**15**	8.92 (0.09)	8.12 (0.09)	7.45 (0.10)	0.20 (0.13)	1.6	–0.56 (0.15)	0.3	0.76 (0.15)*	5.7
**16**	8.90 (0.19)	8.52 (0.19)	7.84 (0.07)	0.21 (0.20)	1.6	–0.14 (0.22)	0.7	0.35 (0.13)	2.2
***n*^Δ^**	1.49 (0.21)	1.28 (0.14)	1.14 (0.06)						


*The symbol “^∗^” indicates significant difference from CP 55,940 for the same bias comparison; significance levels as defined in the methods. ^Δ^“*n*” is the transducer function slope which was determined for each signalling pathway as a shared constraint between ligands when fitting the operational model.*

As expected from the above exploration, compounds **16** and **10** did not demonstrate any indication of between-pathway bias. On the other hand, **7** exhibited statistically significant bias in comparison with CP 55,940 when the pERK pathway was compared with cAMP (*p* < 0.0001, BF 20.3-fold cAMP > pERK) or internalisation (*p* = 0.0001, BF 15.4-fold internalisation > pERK). There was no bias of **7** between cAMP and internalisation (*p* = 0.91). This, combined with the strikingly partial nature of pERK activation, likely indicates a bias of **7** away from activation of pERK, though as noted above this bias seems to be reinforced by this compound’s apparent ability to induce highly efficient coupling of hCB_2_ to internalisation and cAMP inhibition. Meanwhile, compounds **15** and **14** induced cAMP versus pERK with equivalent balance to CP 55,940, however were biased towards internalisation when compared with either of the other two pathways (three of four statistical comparisons to CP 55,940 were statistically significant; **15** was not statistically different from CP 55,940 in cAMP > internalisation, however a significant difference was found between **15** ΔΔlogRs for cAMP > internalisation versus balanced pathway comparison cAMP > pERK, *p* = 0.034).

## Discussion

In this study, thirteen 2,4,6 trisubstituted 1,3,5-triazine derivatives, comprising five previously reported compounds and eight novel compounds, were synthesised and characterised for their affinity and activity at hCB_2_.

Comparison of competitive orthosteric radioligand binding revealed that in all three cases an adamantanyl group was highly favoured over a cyclopentyl group when other substituents were held the same (**7** vs. **2**, **12** vs. **5**, and **13** vs. **6**). This trend was also observed in previous reports (e.g., Yrjölä et al., 2015), where adamantanyl derivatives were more potent in a GTPγS assay than the corresponding cyclopentyl triazine derivatives in all examples reported.

Compounds **7**, **15**, and **16** have previously been published as potent hCB_2_ agonists (compounds “8,” “6,” and “9” from Yrjölä et al., 2015), however no hCB_2_ binding data was reported. The highest affinity compound in our study (**16**) differed from the next most potent (**15**) by the presence of a fluoroethyl instead of methyl group on the piperazine nitrogen, which conferred a nearly half-log increase in affinity. Piperazinyl-**7** had approximately 100-fold lower affinity than methylpiperazinyl-**15**, despite the only difference between these compounds being hydrogen versus methyl on the piperazine nitrogen. Along with the methyl versus hydrogen size difference, a reason for this significant change in CB_2_ binding might be because **7** is likely ionised to a much higher extend at physiological pH in comparison with **15**. We tested seven adamantanyl-ethoxy-triazines with varying linkers from the piperazine ring with a view to identifying linker derivatives which retain affinity and functional activity. We found that incorporation of a hexyl chain (**9**, **12**) was not well tolerated in this position and considerably reduced (though did not completely prevent) binding to hCB_2_, whereas use of a PEG2 linker (**10**, **13**, **14**) was more successful in retaining hCB_2_ affinity.

While we did not investigate binding of these compounds to hCB_1_ in depth, **15** and **16** were indicated to be somewhat CB_2_-selective in that these were relatively high affinity at CB_2_ and a high concentration only partially displaced [^3^H]-CP 55,940 at CB_1_. The prior report of these compounds indicated approximately 1000 to 10,000-fold functional GTPγS selectivity for CB_2_ versus CB_1_ (Yrjölä et al., 2015). None of the other compounds tested (**7**, **10**, **13**, **14**) exhibited considerable orthosteric binding to CB_1_ at 10 μM indicating that these are likely also CB_2_-selective.

We then proceeded to investigate the function of **7**, **10**, **13**, **14**, **15**, and **16** at hCB_2_. Firstly we made a few general observations regarding potency shifts between assays for the compound series. Internalisation tended to exhibit the greatest potencies, typically approximately 0.4 log units higher than for cAMP (other than two exceptions discussed below). pERK usually had the lowest potencies of the three signalling assays tested; 0.8 log units lower than cAMP on average. Given that the cAMP and pERK pathways were measured at very similar time points (5 and 4 min, respectively) and under equivalent assay conditions, it seems unlikely that the reduced pERK potencies were indicative of different degrees of binding occupancy at this time point, and instead may point to lower coupling efficiency of hCB_2_ to this pathway. Alternatively, or perhaps in addition, this could be indicative of a large capacity for activation and detection of ERK phosphorylation in comparison with cAMP flux and hCB_2_ internalisation in our model system. Indeed, our measured efficacies in the pERK pathway were extremely small relative to our positive control PMA-induced response (up to ∼10%) and we have previously measured approximately four times greater Gα_i_ receptor-mediated pERK responses in a HEK hCB_1_ cell line (Finlay et al., 2017). Furthermore, substantial receptor reserve in the cAMP pathway has been reported previously, wherein fewer than 50% of receptors were apparently required to be occupied in order to produce a 50%-maximal response therefore manifesting in high potency responses (Finlay et al., 2017). That study was performed in an analogous model system to ours; HEK cells expressing a lower concentration of hCB_1_ than the expression level of hCB_2_ in the HEK cell line utilised in this study, therefore our hCB_2_ cell line could exhibit even more receptor reserve than the earlier-studied CB_1_ cell line. CP 55,940 was measured to have the highest binding affinity of the compounds tested, acted as a full agonist in all three signalling pathways, and exhibited the smallest range of activation potencies between the pathways, all of which support selection of CP 55,940 as a reference ligand for comparison with previously uncharacterised compounds (van der Westhuizen et al., 2014).

The highest affinity triazine, **16**, had a very similar affinity and functional potencies to CP 55,940 with no evidence of biased agonism. Despite this, **16** appeared to act as a partial agonist in all functional assays we investigated (∼80% of maximum effect measured across all compounds), and this was consistent with prior published GTPγS data (∼70% of maximum measured activity, Yrjölä et al., 2015). This may imply that although the **16** hCB_2_ binding pocket clearly overlaps that for CP 55,940, the spectrum of hCB_2_ conformation(s) facilitated by **16** binding could be slightly different, perhaps resulting in the receptor spending proportionally less time in active conformation(s).

The next highest affinity compound studied, **15**, acted as a full agonist in all pathways measured (including a previously published GTPγS assay, Yrjölä et al., 2015). Therefore, while the presence of a methyl group (**15**) instead of a fluoroethyl (**16**) on the piperazine nitrogen resulted in a somewhat lower affinity, the methyl group in **15** was more conducive to producing full efficacy. Interestingly, although **15** did not show any indication of bias between activation of cAMP and pERK (relative to CP 55,940), the potency of **15** for inducing internalisation was greater than expected and this culminated in significant pathway bias towards internalisation. **14** had the lowest affinity of all the compounds studied and as such parameter measurements were less definitively identified, however the profile of **14** seemed most similar to **15** and bias towards internalisation was also indicated. It therefore seems likely that these compounds facilitate stabilisation of hCB_2_ in conformation(s) that promote internalisation without considerable influence on acute cAMP or pERK pathways. Although receptor activation is typically considered a prerequisite for internalisation, this is not necessarily G protein dependent, as has previously been shown for CB_2_ (whereby receptor internalisation was not PTX-sensitive, Atwood et al., 2012), as well as for the μ-opioid receptor [where morphine barely internalises the receptor but still induces G protein-gated inwardly-rectifying potassium (GIRK) channel activation, Bradbury et al., 2009]. As such, it is not surprising that these pathways could be modulated independently. Given that desensitisation and phosphorylation are generally accepted prerequisites for the onset of internalisation it might have been predicted that the time courses of cAMP inhibition and pERK activation may have been altered (e.g., earlier or more rapid desensitisation), however we did not observe any such change over the time courses monitored. Alternatively, this bias may have been made evident by the more chronic nature of the internalisation assay (measured at 1 h) in comparison with the cAMP and pERK assays (5 and 4 min, respectively). For example it was recently demonstrated that the direction of apparent agonist bias can be reversed depending on the time point assayed and that this may be associated with ligands’ dissociation kinetics (Herenbrink et al., 2016). In other words, drugs with slower off rates may produce bias for pathways assayed at later time points, as receptor occupancy will be higher. Regardless of mechanism, as arrestin recruitment is frequently associated with receptor phosphorylation and internalisation (Krupnick and Benovic, 1998) it would be pertinent to investigate arrestin recruitment with these ligands in future studies. It would also be particularly interesting to investigate whether the increased propensity to internalise the receptor has any consequence on medium- to long-term cannabinoid responsiveness; for example, non-canonical signalling from endosomes might be influenced, and modulation of receptor recycling versus degradation would influence cellular re-sensitisation.

In comparison with CP 55,940, **10** exhibited high potencies in all three signalling pathways relative to its binding affinity. That is, this ligand was seemingly able to induce maximal functional effects while occupying fewer receptors than CP 55,940. The simplest explanation for this phenomenon is that once bound to hCB_2_ **10** is more effective at stabilising the active conformation(s) of hCB_2_ than CP 55,940. This would imply that “receptor reserve” must be present for all three signalling pathways we studied, and the high intrinsic activity of compound **10** manifests as leftward potency shifts relative to receptor occupancy. Ligand **10** therefore has the potential to be a greater efficacy hCB_2_ agonist than CP 55,940 if tested in a model system with sufficiently low hCB_2_ expression and/or large capacity for generating/reporting signalling responses (Luttrell, 2014). For internalisation, measuring an earlier time point may also have been illuminating in this regard. This theory would certainly be interesting to verify in a follow-up study. It is also plausible that this ligand possesses a relatively rapid hCB_2_ on-rate which manifests as greater assay potency due to more efficient acute binding of **10** to hCB_2_ in comparison with the other ligands tested. Although we did not detect differences in the onset of cAMP or pERK signalling between any of the ligands studied (which could have supported this theory), it is possible that either our assays were not sufficiently temporally sensitive to reveal such a difference, or the rates of ligand-receptor association for the range of ligands tested were all sufficient to reach the maximum rate of pathway activation for our model system. An additional hypothesis could be that an increased local concentration of ligand at the plasma membrane – for example if the membrane was acting as a reservoir or “sink” (Vauquelin, 2016) – might manifest as high apparent intrinsic coupling efficiency. However, this ligand was not predicted to have notably unique physicochemical properties in the context of the other triazines we pharmacologically characterised, and given the increased polarity in comparison with “traditional” cannabinoids would be less likely to accumulate in this manner than our reference ligand, CP 55,940. Taken together, it seems that **10** acts as a balanced high intrinsic efficacy agonist for activating hCB_2_ in the signalling pathways we studied.

**7** exhibited a particularly interesting signalling profile. In our model system, **7** was a full agonist and produced a similar potency:affinity profile to high intrinsic efficacy agonist **10** in the cAMP and internalisation assays suggesting that it too likely acts with high intrinsic efficacy in these pathways. However, it was a weak partial agonist in the pERK pathway, producing only ∼36% of the maximal measured response across all ligands and exhibiting significant bias away from pERK relative to CP 55,940 and the other two signalling pathways measured. This is somewhat in agreement with previously reported data wherein **7** elicited ∼60% of maximum measured activity in a GTPγS assay (Yrjölä et al., 2015). While this bias is exciting to identify and has the potential to produce unique downstream functional effects in comparison with balanced ligands, the mechanism driving this bias is not immediately obvious. In particular, partial agonism in GTPγS and PTX-sensitivity of ERK phosphorylation do not reconcile well with high intrinsic activation of Gα_i/o_-mediated adenylate cyclase inhibition, although similar bias towards adenylate cyclase inhibition in comparison with pERK and GTPγS has been observed for opioid ligands activating the μ-opioid receptor (Thompson et al., 2015). Clearly more in-depth study of this ligand is warranted in order to further probe the mechanism producing this pattern of signalling bias at hCB_2_. However, to our knowledge this ligand is unique in that it represents the first identification of a hCB_2_ agonist with bias away from pERK, whereas two prior studies have identified ligands with bias towards pERK versus cAMP (Shoemaker et al., 2005; Soethoudt et al., 2017). Given the highly context-dependent nature of activation of the ERK pathway on cellular function the potential clinical relevance of a ligand with reduced propensity to stimulate ERK phosphorylation such as **7** is difficult to predict. Nonetheless, **7** would certainly be an interesting candidate for *in vivo* study to determine functional effects in comparison with un- or differentially-biased ligands, which may well be illuminating in efforts towards development of CB_2_-targeted therapeutics.

The most intriguing findings of our study came about in the functional characterisation of **13** which has a primary amine at the end of the PEG2 linker. Surprisingly this behaved extremely differently to all the other compounds tested, acting as a neutral antagonist in the internalisation pathway, an unconventional transient inverse agonist for adenylate cyclase inhibition, and an extremely weak partial agonist for pERK activation. Note that despite appearing to act as a neutral antagonist in the internalisation pathway, inverse agonism wouldn’t necessarily be able to be detected at this time point in this assay (which would have manifested in this assay as an apparent increase above the vehicle-treated condition, as described in Grimsey et al., 2011), nor perhaps extremely weak partial agonism. We therefore cannot conclude whether this compound is acting as an inverse agonist, neutral antagonist, or very weak partial agonist in this pathway. While examples of compounds acting as both an agonist and antagonist via different signal transducers at the same receptor have been reported (e.g., Kilts et al., 2002; De Deurwaerdere et al., 2004; Corbisier et al., 2017), including for CB_2_ (Schuehly et al., 2011; Dhopeshwarkar and Mackie, 2016), the fact that the responses in both signalling assays were PTX-sensitive (and thus both downstream of CB_2_ coupling to Gα_i_) is tantalisingly conflicting, as it implies that this compound is acting as both a weak agonist and inverse agonist at CB_2_ via the same G protein.

The cAMP kinetic signature of **13** was also unique in that inverse agonism was very transient, in contrast with a typical inverse agonist (SR 144528) which exhibited sustained inverse agonism over the entire time course of the assay. It is vaguely feasible, particularly given the fairly low potency of **13**, that this interesting functional pattern could have been produced as a result of the presence of trace impurity present in our synthesised **13** sample, however, the identity of **13** was fully characterised by NMR and HRMS, and the purity of the sample was 100% by analytical HPLC (as described in the section “Chemistry”). Relatedly, **13** exhibited no apparent non-CB_2_-mediated effects in HEK cells in the pathways we measured. One explanation for the transient inverse agonism could be instability of **13** in assay buffer, however this seems highly unlikely given the time course of cAMP inverse agonism was unaffected by different lengths of time in solution prior to the start of the assay (data not shown). The primary amine of **13** is a nucleophilic functional group however it would be highly ionised at physiological pH, thereby making reaction with an electrophile unlikely. Furthermore, there is no apparent strong electrophile present in the cAMP assay conditions, nor anything that would appear different in these terms between the different signalling pathway assays. While there is potential for metabolism of any of the compounds tested **13** does not stand out as being more likely to act as a substrate than the others. It is also unlikely that **13** could be undergoing *N*-glucuronidation given HEK cells are usually found to lack glucuronosyltransferase activity (e.g., Southwood et al., 2007). We therefore conclude that the kinetic signature observed for **13** is highly likely to be a genuine characteristic of its effect on CB_2_.

The authors are not aware of findings in the literature analogous to this transient inverse agonism (although it should be noted that to date very few studies have utilised time courses when monitoring inverse agonism, and so transient responses might not have been detected even if present). However, we have postulated some theories with potential to explain the unique functional profile of **13**, which would be interesting to address in future studies. One hypothesis is that **13** is acting as a bitopic ligand, i.e., it has affinity/activity for both orthosteric and allosteric sites (Kamal and Jockers, 2009), a phenomenon which has previously been reported for the muscarinic receptors (Steinfeld et al., 2007; Valant et al., 2008). Indeed, we have previously reported CB_1_ allosteric modulators to have unique temporal influences on inhibition of cAMP production (Cawston et al., 2013, 2015). Given that **10** and **14** possesses a similar PEG2 linker as **13**, the PEG2 linker in itself seems unlikely to be primarily responsible for exerting the observed functional pattern, although the presence of an acetyl versus *tert*-butoxy versus primary amine at the linker terminus would likely have an effect on ligand-receptor interactions. Whether via orthosteric-induced conformational changes or allosterism it is indeed feasible that small changes in ligand structure can correspond to major structural movements at the cytosolic face of the receptor and consequently affect signalling considerably (Shonberg et al., 2013). Alternatively, or perhaps in concert with this theory, the unique cAMP temporal fingerprint could arise as a consequence of the small ERK activation (or activation of other signalling pathways not yet investigated) inducing a downstream effect such as stimulating a post-translational modification (PTM) on CB_2_. Following initial stabilisation of a predominantly inactive conformation by **13** (notwithstanding extremely weak pERK activation), perhaps such a PTM could shift the conformational equilibrium away from the inactive state and facilitate the receptor re-gaining the ability to inhibit adenylate cyclase, thereby producing the apparent loss of inverse agonism and return to constitutive inhibition of adenylate cyclase. GPCR constitutive activity has been shown to increase by mutation of particular residues (Pauwels and Wurch, 1998), as such it is feasible that PTMs of specific residues could also induce the same effect. Similarly, influences on dimerisation or other protein–protein interactions (e.g., Glass and Felder, 1997; Bakker et al., 2004), or receptor transit between membrane microdomains (e.g., Jiao et al., 2005), may be involved. Another possible factor in this unique signalling fingerprint is the physicochemical properties of **13**. Of the compounds we characterised functionally, **13** was calculated as having the most polar cLogP and cLogD_7.4_. Polar compounds may have an alternative mode of access to hCB_2_ compared to more lipophilic cannabinoids which likely enter via the lipid bilayer (Hurst et al., 2010). **13** could perhaps therefore have a different mode of access to the receptor compared with the remainder of the compounds. It is also tempting to hypothesise that, due to its increased polarity, **13** might not have access to intracellular CB_2_ or that access may be relatively slow. If intracellular CB_2_ usually contributes to signalling when cell-permeable ligands are utilised, an inability or delay to activating intracellular CB_2_ may profoundly influence the apparent signalling profile. Of course, there are many factors that contribute to the cellular permeability of a compound, degree of polarity and ionisation being just two. It would certainly be interesting to directly investigate cell permeability of **13** in future, potentially utilising assays such as the parallel artificial membrane permeability assay (PAMPA). Finally, physicochemical properties might also influence ligand partitioning within the plasma membrane (e.g., lipid rafts) and thereby perhaps **13** could interact with a unique subset of CB_2_ and/or a changing subset over time (Barnett-Norris et al., 2005; Vauquelin and Packeu, 2009).

In this study we functionally characterised a set of thirteen 2,4,6-trisubstituted 1,3,5-triazines, all of which are considerably more polar than most cannabinoids studied to date, and eight of which were novel. Compounds **7**, **14** (UOSD017) and **15** exhibited interesting bias profiles (**7** biased away from pERK activation, **14** and **15** biased towards internalisation) and one compound exhibited high potency in all signalling pathways measured relative to affinity (**10** [UOSD015]). Meanwhile, **13** (UOSD008) produced a completely unique functional profile at hCB_2_, acting as a mixed agonist/inverse agonist. It will be interesting to further characterise the functional activity of these ligands and investigate the mechanisms underlying these apparent biases in future studies. **16** was found to have a similar bias profile to that of CP 55,940 (whereby it was relatively balanced across all pathways measured) however, due to its greater polarity, would likely have a more favourable pharmacokinetic profile. It would accordingly be particularly interesting to compare the activity of these compounds *in vivo*. Some of our novel compounds included the incorporation of linkers for potential secondary reporter or fluorophore attachment, but require further optimisation since none of our novel compounds rivalled the affinity of **15**, although **10** (UOSD015) which includes a Boc-protected PEG2-piperazinyl moiety is a promising candidate for further modification. This study has characterised a number of CB_2_ ligands with greater polarity than traditional cannabinoid agonists and that exhibit moderate to high affinity and unique signalling patterns. As such, these could form the basis of a ligand “toolbox” for further CB_2_ *in vitro* and *in vivo* studies, and potentially be useful in the development of peripherally-restricted cannabinoid ligands.

## Author Contributions

CO contributed to the experimental and project design, carried out the majority of the experiments and analysis, wrote the first draft of the manuscript, and prepared all figures and tables. SdlH synthesised and purified the compounds. YS carried out the pERK experiments and associated analysis. MG contributed to project design and interpretation. AV designed the novel compounds, supervised the synthesis, and contributed to project design and interpretation. NG designed the project and analysis, oversaw all elements, and obtained the funding. All authors reviewed and edited the manuscript.

## Conflict of Interest Statement

The authors declare that the research was conducted in the absence of any commercial or financial relationships that could be construed as a potential conflict of interest.
