# Erratum: Cannabinoid Receptor 2 Signalling Bias Elicited by 2,4,6-Trisubstituted 1,3,5-Triazines

**DOI:** 10.3389/fphar.2019.00418

**Published:** 2019-04-05

**Authors:** Frontiers Production Office

**Affiliations:** Frontiers Media SA, Lausanne, Switzerland

**Keywords:** cannabinoid receptor 2 (CB_2_), G protein-coupled receptor, signalling bias, signalling, synthetic cannabinoid, drug design, medicinal chemistry, immune therapeutics

Due to a production error, 24 references with more than six authors were listed in the Reference list without “et al.” The references affected are Bouaboula et al. (1993, 1996); Felder et al. (1995); Galiegue et al. (1995); Favata et al. (1998); Portier et al. (1999); Kilts et al. (2002); Karsak et al. (2005); Wright et al. (2005); Ofek et al. (2006); Jiang et al. (2007); Valant et al. (2008); Cencioni et al. (2010); Hurst et al. (2010); Schuehly et al. (2011); van der Stelt et al. (2011); Kleyer et al. (2012); Odan et al. (2012); Shonberg et al. (2013); Brailoiu et al. (2014); Herenbrink et al. (2016); Finlay et al. (2017); Soethoudt et al. (2017) and Yrjölä et al. (2015).

The publisher apologizes for this mistake. The original version of this article has been updated.
